# Detection of Lipid-Mediated Diffused Pathological Changes in Multiple Sclerosis

**DOI:** 10.21203/rs.3.rs-10730332/v1

**Published:** 2026-08-28

**Authors:** Blake Benyard, Dushyant Kumar, Neil E. Wilson, Anshuman Swain, Sunil Kumar Khokhar, Paul Jacobs, Narayan Datt Soni, Matthew K Schindler, Mohammad Haris, Ravinder Reddy

**Affiliations:** University of Pennsylvania; University of Pennsylvania; University of Pennsylvania; University of Pennsylvania; University of Pennsylvania; University of Pennsylvania; University of Pennsylvania; University of Pennsylvania; University of Pennsylvania; University of Pennsylvania

## Abstract

**Background:**

Lipid dysregulation and myelin breakdown are hallmark features of multiple sclerosis (MS), yet current noninvasive biomarkers have limited sensitivity for detecting both focal demyelination and diffuse microstructural injury. We evaluated transient nuclear Overhauser effect (tNOE) MRI, a lipid-sensitive imaging technique, for mapping myelin-related alterations in MS.

**Methods:**

tNOE-MRI was performed in four MS patients and four healthy controls using a 7T scanner equipped with a 32-channel receive phased-array coil. Scan reproducibility was assessed across repeated sessions, and tNOE signal patterns were analyzed in major white-matter regions, including corpuscallosum and internal-capsule.

**Results:**

tNOE maps demonstrated strong contrast in highly myelinated structures, like splenium of corpuscallosum and internal-capsule. Inter-day and intra-day coefficients of variation were < 5%, indicating high measurement consistency. Quantitative analysis revealed that MS patients had significantly lower tNOE contrast in white-matter (12.6%vs16.1%) and grey-matter (5.9%vs7.2%) compared to healthy controls. The splenium exhibited the greatest tNOE reduction (~ 36%) in MS.

**Discussion:**

Reduced tNOE contrast corresponded with known regional differences in myelin density and suggests sensitivity to myelin-integrity. Lower tNOE signals in both white and gray matter indicate diffuse tissue alterations beyond focal lesions. With an acquisition time of ~ 3 minutes, tNOE-MRI shows promise as a rapid biomarker for MS diagnosis.

## Introduction

Multiple sclerosis (MS) affects more than 2.8 million people worldwide^1^, including approximately 1 million in the United States^2^, making it one of the leading causes of non-traumatic neurological disability in young adults. Most patients present with relapsing-remitting MS (RRMS), characterized by acute relapses and partial recovery, while about 10–20% develop primary progressive MS (PPMS), marked by steady neurological decline^31^. Over the past two decades, disease-modifying therapies (DMTs) such as interferon-β, fingolimod, and ocrelizumab have dramatically reduced relapse rates and new lesion formation in RRMS^4–6^. Nevertheless, many patients, particularly those with PPMS, continue to accumulate disability despite effective lesion control, underscoring the contribution of diffuse and progressive pathology that remains poorly understood^7,8^. This disconnect underscores the contribution of diffuse and progressive pathology, driven in part by widespread myelin membrane disruption and lipid imbalance, processes that are not adequately captured by conventional lesion-based MRI.

Conventional MRI techniques such as T_2_-FLAIR, diffusion imaging, and magnetization transfer ratio (MTR) provide valuable markers of demyelination and axonal injury, yet they lack biochemical specificity^9^. While T_2_ hyperintense lesions reflect demyelination and inflammation, they do not directly quantify lipid loss^10–13^. Traditional approaches to lipid imaging, such as Dixon imaging, inversion-recovery fat suppression, and chemically selective saturation, lack sensitivity in the brain due to the inherently short T_2_ of lipid protons^14^. Relaxometry-based methods, such as myelin water imaging^15^, can provide indirect estimates of myelin content by isolating signal from protons trapped between myelin bilayers; however, these approaches may be less sensitive to early demyelination and lipid alterations that precede overt structural loss.

To overcome this limitation, magnetization transfer (MT)-based approaches have been proposed^16^. Steady-state nuclear Overhauser effect (ssNOE) imaging has demonstrated sensitivity to lipid protons in vivo, with NOE signals originating from methyl and methylene groups resonating within 2–4 ppm upfield of water^17–24^. However, ssNOE is limited by long acquisition times, contamination by direct saturation and MT effects, and difficulties in disentangling these confounds in the presence of B_0_ and B_1_ inhomogeneities^23^. These challenges have hindered its validation as a reliable marker of brain lipid content.

Recently, transient NOE (tNOE) methods have emerged as a promising alternative, offering enhanced specificity by isolating cross-relaxation effects from aliphatic protons while reducing MT contamination^25^. Our group has demonstrated the feasibility of measuring tNOE contributions from lipids and other macromolecules at 7T using frequency-selective inversion recovery with sinc pulses. While 7T MRI provides superior SNR and spectral resolution, its poorer B_1_ homogeneity complicates quantitative analysis^26–29^. This is particularly problematic when mapping pathological lipid loss in diseases such as MS, where sensitivity to subtle changes is essential.

In this study, we advanced tNOE imaging by developing a 3D acquisition protocol at 7T and evaluating its robustness and repeatability in healthy controls. We then applied the technique to MS patients. Our findings show that tNOE imaging is sensitive to regional lipid changes within MS lesions, as detected by conventional clinical scans. Additionally, tNOE consistently identified diffuse white matter changes outside of lesions in MS patients, alterations that were not visible using standard clinical protocols. These results establish tNOE MRI as a promising new biomarker for investigating lipid changes associated with MS pathology and provide a foundation for future lipid-sensitive neuroimaging studies in aging and neurodegenerative diseases.

## Methods

Transient NOE imaging was performed on a BRAINO phantom (GE Medical Systems, Milwaukee, WI, USA), five healthy participants (5 males), and four patients with MS on the 7T whole body scanner (MAGNETOM Terra, Siemens Healthcare, Erlangen, Germany) using a single-channel transmit / 32-channel receive phased-array proton head coil (Nova Medical, Wilmington, MA, USA). The MS patients (two male, two female), all with relapsing remitting, were recruited between June and August of 2025 from the University of Pennsylvania’s Department of Neurology, MS and Related Disorders Outpatient Clinic. All MS participants met the 2017 McDonald’s Criteria for dissemination in time and space^30^. Three of the participants were on a disease-modifying therapy, while one was not. All human data were acquired under an approved University of Pennsylvania Institutional Review Board Protocol after obtaining written informed consent. The average age of the MS participants was 39.2 years (27–65 years), and the average age of the healthy participants was 35.3 years (22–47 years).

tNOE imaging utilizes a magnetization-preparation module that employs a frequency-selective inversion pulse (42.6 ms HS adiabatic pulse covering a 3 ppm bandwidth) applied to the aliphatic protons of lipids (−3.5 ppm). The HS pulse was followed by a variable mixing time (τ) that is followed by a multi-shot turbo-Flash (TFL) readout with centric phase-encoding order having a TR = 3.5 ms, TE = 1.47 ms, receiver bandwidth = 550 Hz/pixel. Imaging parameters were as follows: in-plane resolution = 1 mm × 1 mm, slice thickness = 5 mm, matrix size = 240 × 240 x 12, averages = 2, TR = 5 s, tfl-TE = 1.62 ms, tfl-TR = 3.5 ms, τ = 150 ms, and flip angle = 4°. One slice at both ends of the imaging slab was discarded to avoid phasing artifacts. Two intra-day and three inter-day scans were performed on healthy subjects to assess repeatability at 7T. To obtain consistent slice locations across subjects, we used an in-house-developed co-registration program. Brain subregions: splenium of corpus callosum (SCC), genu of corpus callosum (GCC), internal capsule (IC), posterior thalamic radiation (PTR) and corona radita (CR) were obtained via automated segmentation of an acquired MP2RAGE image, with the following steps: 1) bias field correction using Advanced Normalization Tools^31^ ; 2) brain masking using HD-BET^32^ ; 3) segmentation for subcortical gray matter regions using FSL FIRST^33^.

The water inversion efficiency maps were calculated using Eq. 1:

[1] $$\mathrm{Water Inversion Efficiency}\;(\%)=100*\frac{S\;(0\mathrm{ppm})}{S\;(100\mathrm{ppm})}$$

In this expression, S (0ppm) and S (100ppm) are the signal intensities with the inversion pulse applied at 0 ppm and 100 ppm, respectively.

Data processing was performed using in-house MATLAB (version R2019a) scripts. The tNOE contrast was calculated using Eq. 2:

[2] $${\mathrm{tNOE}}_{\mathrm{MTR}}\;(\%)=100*\frac{S\;(100\mathrm{ppm})-S\;(-3.5\mathrm{ppm})}{S\;(100\mathrm{ppm})}$$

In this expression, S(100ppm) and S(− 3.5ppm) are the signal intensities with the inversion pulse applied at the frequency offset of 100 ppm, and − 3.5 ppm, respectively.

The intra- and inter-subject coefficients of variation (CoV) for each volunteer were calculated as the percent normalized ratio of the standard deviation (σ) of the contrast and mean (μ) of the pixels across the slab, using Eq. 3.


[3] $$\mathrm{CoV}\;(\%)=100*\left(\frac{\sigma }{\mu }\right)$$

## Results

**Figure 1:**
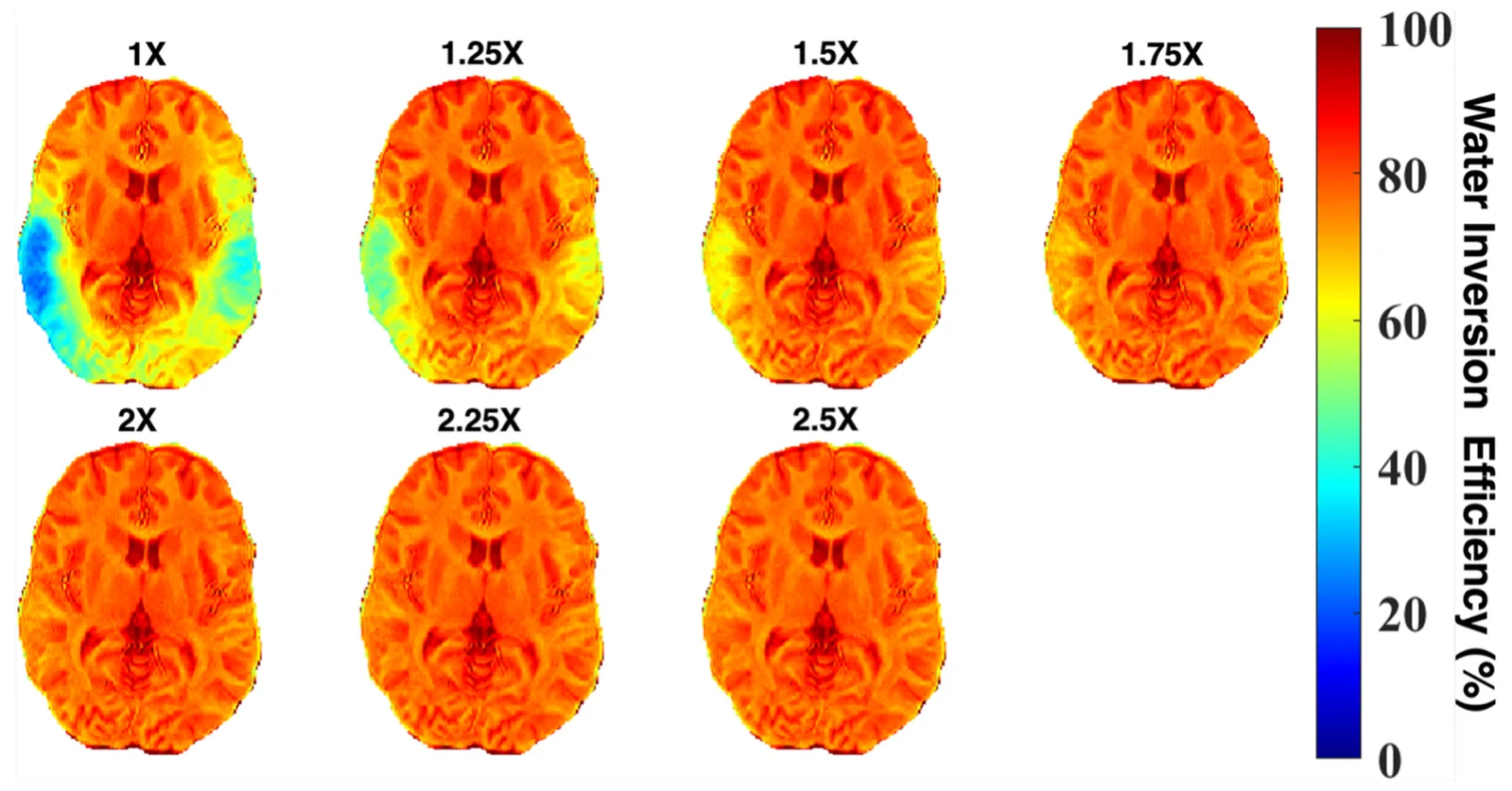
Water inversion efficiency maps acquired on a human subject with multiples of the system-supplied reference voltage (1X). The efficiency of water inversion (%)at twice the recommended voltage (2X)shows strong water inversion and uniformity.

**Figure 2:**
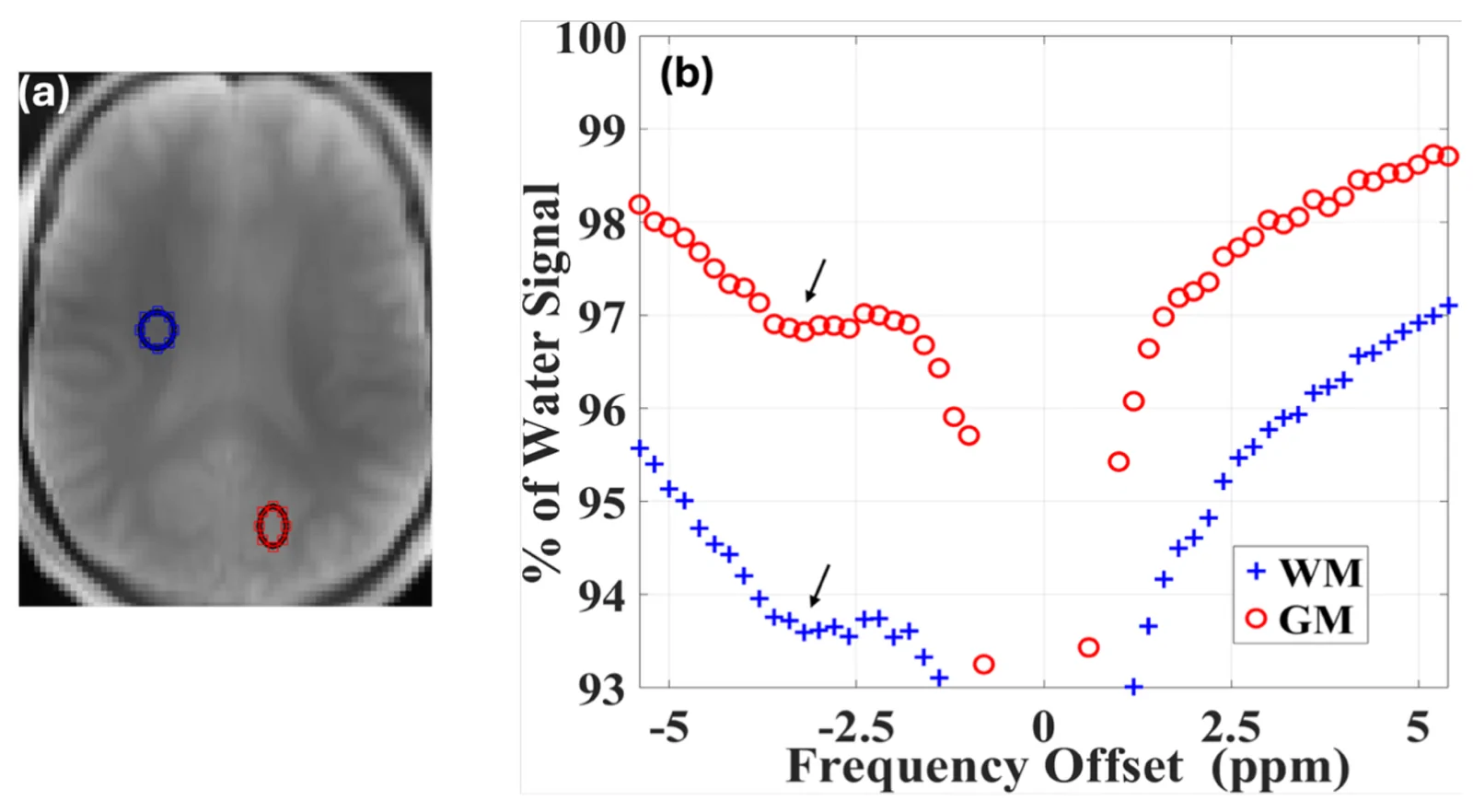
Reference image of one of the subjects with GM (red) and WM (blue) ROIs (a). Inversion recovery spectrum across frequency offsets: −5 to 5 ppm (b). A clear peak in the tNOE signal was observed upfield at 3.5 ppm (arrows).

To achieve optimal conditions for tNOE measurements, validation experiments were conducted using the BRAINO phantom, which closely mimics the T_1_ relaxation properties of the human brain. This phantom served as a reliable test to standardize the protocol before applying it to human subjects. Images were acquired across a range of flip angles to determine the ideal settings for achieving uniform tNOE contrast. Shown in Fig. 1 is a calibration curve from increasing the voltage amplification factor (flip-angle) in the human brain. From these experiments, the optimal transmit voltage for achieving uniform adiabatic conditions across the imaging volume was determined to be twice the system-recommended value, yielding consistent and robust water inversion. After applying twice the system-recommended inversion pulse reference voltage, the inversion plateaus, and the signal remains constant across the slice. Figure 2 illustrates the z-spectra of the tNOE signal across offset frequencies ranging from − 5 to 5 ppm for both GM and WM regions of the brain in one representative subject. The spectrum reveals a distinct dip at 3.5 ppm upfield of bulk water, corresponding to the tNOE signal from lipids. This observation aligns closely with the signal that we see in the conventional steady-state NOE z-spectrum^17^.

**Figure 3:**
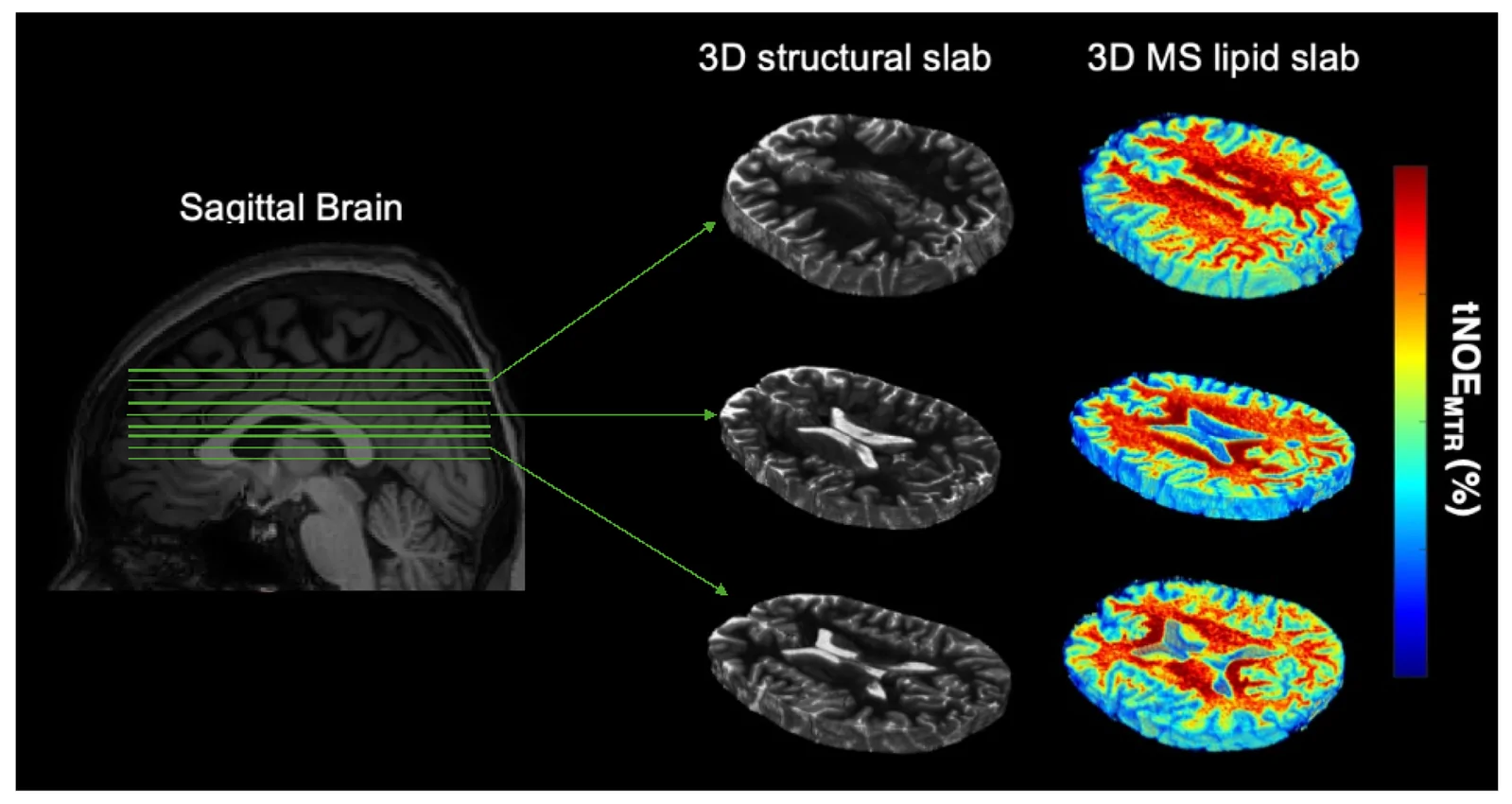
Representative 3D structural and lipid-sensitive tNOE imaging acquired through the corpus callosum in an MS subject. The left panel shows a sagittal T1-weighted brain image with green lines indicating the position and coverage of the partial 3D imaging slab. Corresponding slices from the 3D structural slab (middle column) and lipid-sensitive tNOE maps (right column) are displayed. The tNOE maps, acquired using the optimized adiabatic preparation, demonstrate strong contrast between white matter and surrounding tissues, highlighting regional variations in lipid-related signal across the corpus callosum and adjacent white matter structures. The color bar represents tNOE_MTR_ contrast (%).

**Figure 4:**
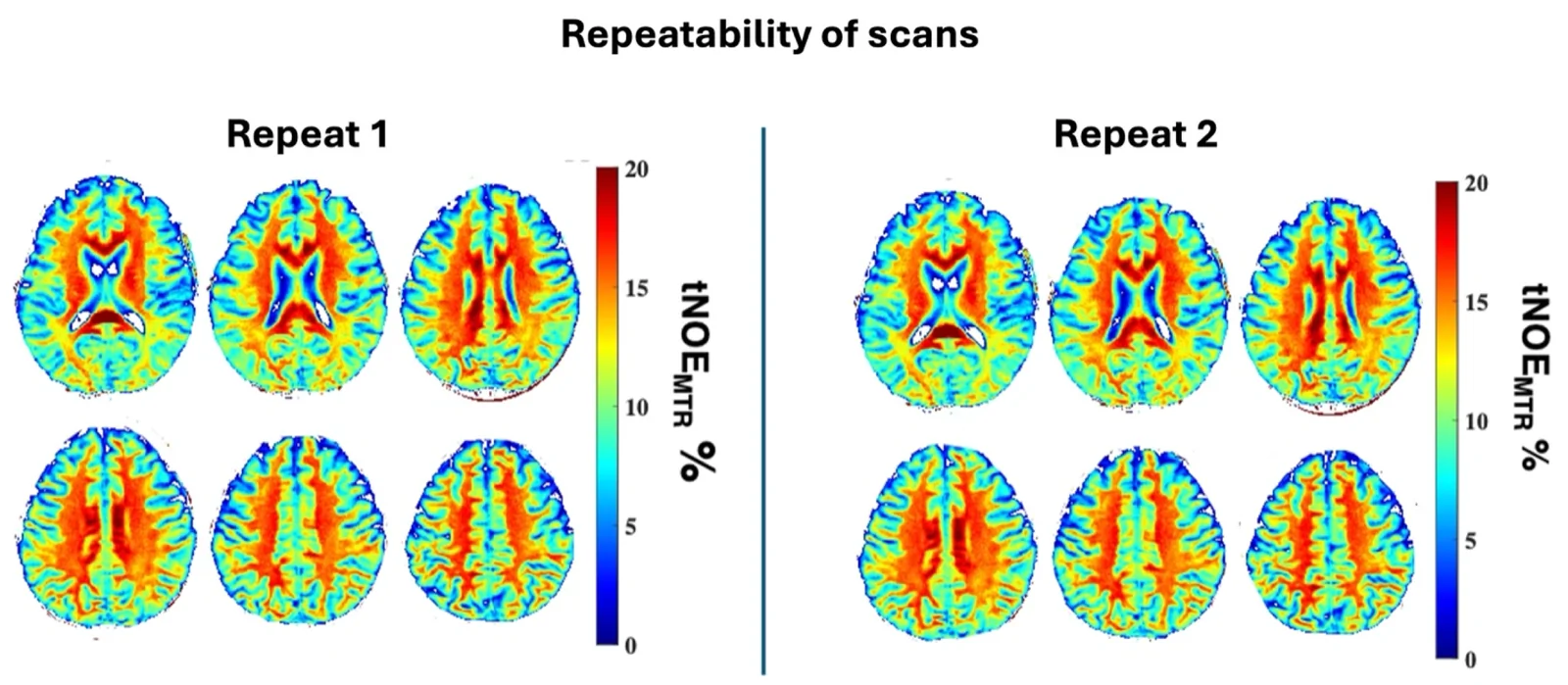
Repeatability assessment of tNOE maps generated from the same subject on two separate days. Consistent contrast between GM and WM regions highlights the reproducibility of the optimized protocol.

The imaging slab was carefully positioned across the corpus callosum to encompass white matter regions known to be particularly vulnerable to demyelination in MS (Fig. 3). Figure 4 presents tNOE_MTR_ maps from one representative subject scanned on two separate days. These maps demonstrate consistent contrast between WM and GM regions, underscoring the repeatability of the measurements. To further evaluate the protocol’s robustness, inter- and intra-day repeatability was analyzed across five subjects (Table 1). Notably, the third subject was scanned one week apart, yielding CoVs of 0.91% in WM and 0.77% in GM. Across all subjects, inter-day and intra-day scans showed CoVs ~ 5%, indicating high measurement consistency. These findings underscore the reliability of the optimized tNOE measurement protocol and its potential for application in clinical and research settings.

**Figure 5:**
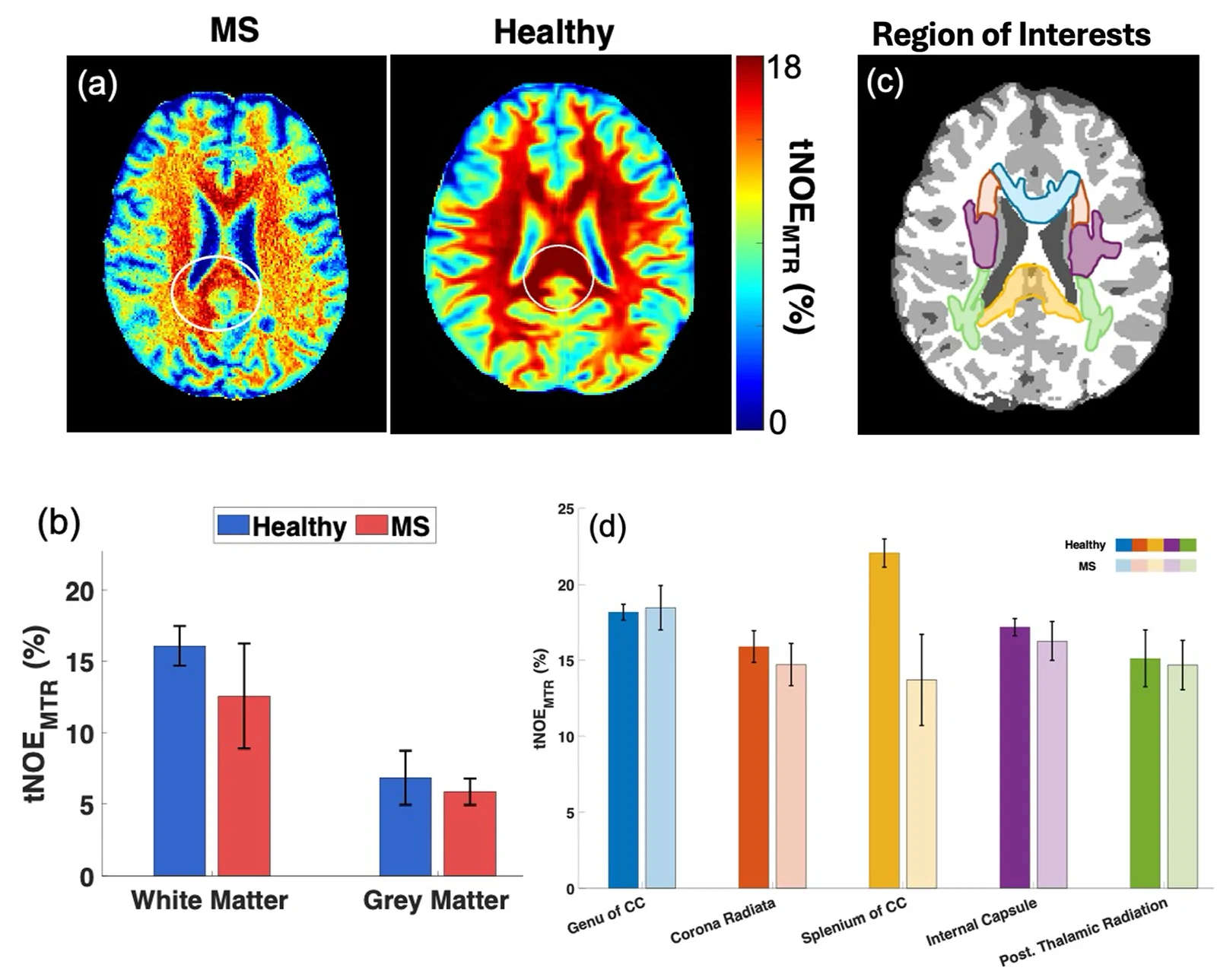
A side-by-side comparison of tNOE_MTR_ maps from a healthy and MS participant (a). A white circle around the splenium region shows a highlighted tNOE contrast in the healthy participant compared with the MS participant. The bar plot summarizes group-averaged tNOE values, demonstrating higher lipid-sensitive signal in healthy participants (16.07 ± 1.38 %) than in MS participants (12.57 ± 3.69 %) in WM, and (7.2 ± 3.4 %) compared to MS participants (5.89 ± 2.33 %) in GM (b). WM subregions overlayed onto a segmentation map of one of the subjects. The GCC (blue), CR (orange), SCC (yellow), IC (purple), and PTR (green) are highlighted (c). Bar plot showing the tNOE_MTR_ values for the WM subregions of healthy and MS participants (d). The splenium exhibited the largest difference in tNOE_MTR_ signal (~8%).

In healthy participants, tNOE maps revealed a strong lipid-associated contrast across various myelinated tracts. Notably, these tracts included the splenium and the internal capsule, regions critical for various cognitive and motor functions. In stark contrast, participants with MS exhibited a marked reduction in signal intensity in these same areas (Fig. 5), suggesting significant alterations in lipid content associated with myelin integrity. Quantitative analysis of the tNOE contrast in white matter demonstrated a significant difference between the cohorts, with MS patients showing an average contrast of 12.6 ± 3.7%, compared to a healthier average of 16.1 ± 1.4% in controls. This finding indicates a reduction in white-matter lipid content in MS patients. Similarly, in grey matter, a comparable pattern was observed, with MS participants exhibiting a mean tNOE contrast of 5.9 ± 2.3%, compared with 7.2 ± 3.4% in healthy participants. This finding underscores the potential for diffuse microstructural changes in the brain that extend beyond the typical focal lesions associated with MS.

Regional analysis pinpointed the splenium of the corpus callosum (SCC) as the most affected tract overall, with a reported median reduction of 36% in lipid-related contrast. In healthy participants, the tNOE contrast in this region was 22.1 ± 0.9%, compared to a considerably lower 13.7 ± 3.0% in MS individuals. Moreover, smaller but consistent reductions were also noted in other critical tracts, including the genu of the corpus callosum, the corona radiata, the posterior thalamic radiation, and the internal capsule. These comprehensive findings illustrate the promise of tNOE MRI for detecting both focal and diffuse lipid alterations that are not apparent on conventional imaging. The ability of tNOE to reveal these subtle changes positions it as a potentially sensitive biomarker for early myelin disruption in MS, with significant implications for diagnosis and monitoring of disease progression.

**Figure 6:**
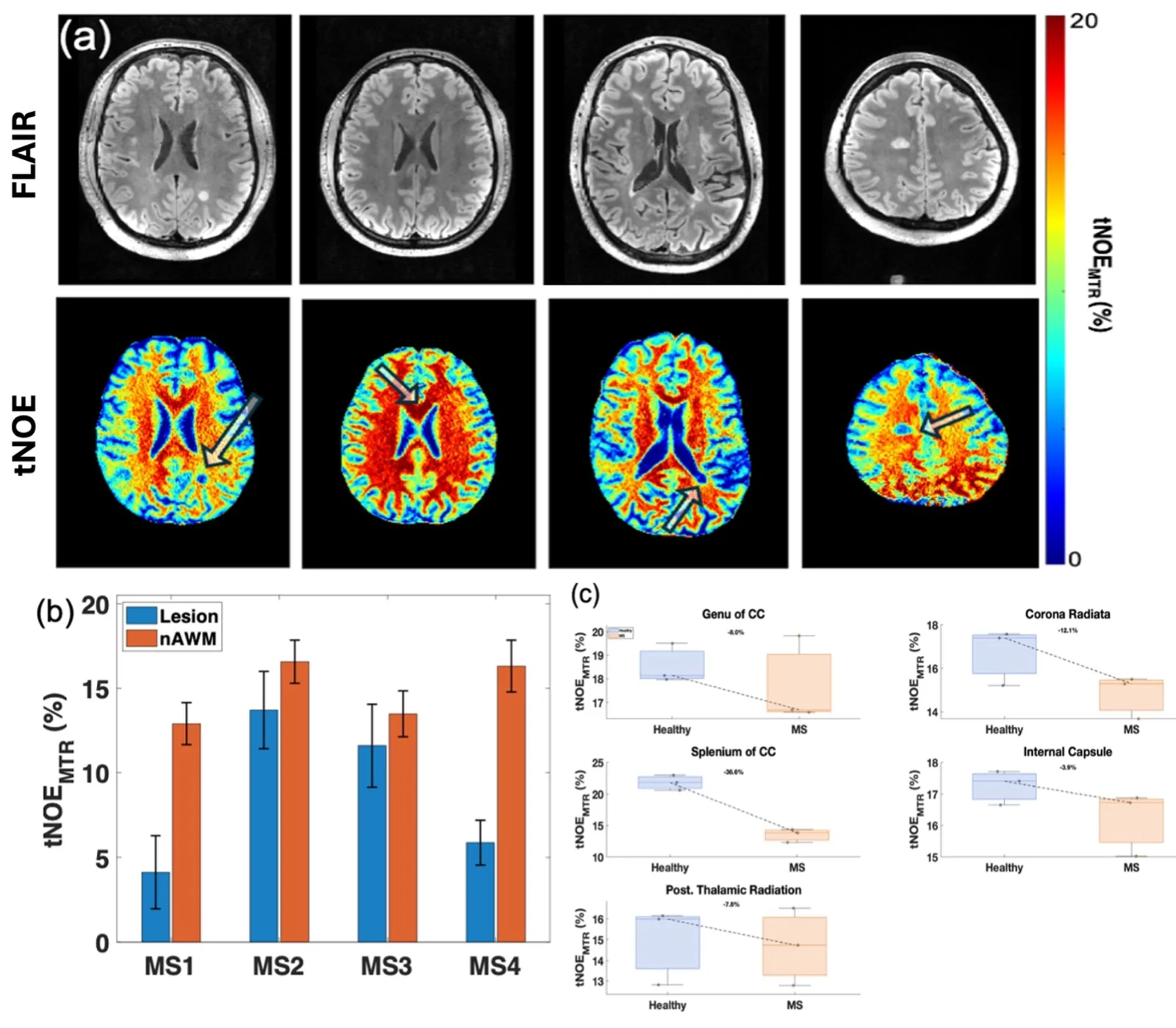
The first column is the FLAIR images of the MS subjects. The second column is the corresponding tNOE maps, where diffuse changes are shown by arrow (a). A barplot of the diffuse changes shows that the average tNOE_MTR_ values (~9%) are lower than the normal appearing white matter (nAWM) (~15%) in all MS subjects (b). Boxplots of tNOE contrast across healthy and MS participants for selected white matter regions (c). Individual subject values are shown as points, with group medians overlaid. Percent differences between groups are indicated above each box. The splenium of the corpus callosum (SCC) exhibited the largest median difference (36%), consistent with the regional substructure analysis in figure 5. Additional differences were observed in the genu of the corpus callosum (GCC, 8%), corona radiata (CR, 12.8%), internal capsule (IC, 4%), and posterior thalamic radiation (PTR, 8%).

Compared with conventional T_2_-FLAIR imaging, tNOE maps revealed subtle, spatially diffuse signal changes extending beyond the borders of visible lesions (Fig. 6). This indicates that conventional imaging might not capture the full extent of the pathology. These alterations likely reflect early or subclinical demyelination in otherwise normal-appearing white matter, which may go unnoticed on standard imaging. When specifically examining mean tNOE values, lesions in MS patients averaged approximately 9.3%, whereas adjacent normal-appearing white matter averaged approximately 15%. This highlights significant changes even in regions that do not appear overtly pathological.

## Discussion

Lipid imaging in the brain has emerged as a critical frontier in neuroscience and clinical research, offering insights into the molecular underpinnings of neurological disorders such as MS^34^. In MS, the breakdown of lipid-rich myelin sheaths underlies axonal dysfunction, neurodegeneration, and ultimately disability progression^35^. Quantitative imaging of lipids, therefore, holds promise as a sensitive biomarker for tracking both overt demyelination and diffuse changes that precede lesion formation.

In this work, we demonstrate the feasibility of partial 3D tNOE-MRI for probing lipid composition in the human brain at 7T. The optimized protocol yielded low coefficients of variation (< 6%) across intra- and inter-day measurements, underscoring its potential utility for longitudinal studies and clinical translation. tNOE maps highlighted regional variability in myelinated tracts, including the corpus callosum, splenium, internal capsule, and posterior thalamic radiation, across healthy participants and MS subjects. Regions such as the splenium of the corpus callosum (SCC), genu of the corpus callosum (GCC), and internal capsule (IC), which are rich in densely packed, highly myelinated fibers, exhibited stronger tNOE_MTR_ signals, likely reflecting a higher relative lipid content. Post-mortem histological analysis of adult human corpus callosum using Luxol Fast Blue (LFB) staining has revealed higher myelin density in posterior regions, such as the SCC, compared to anterior regions (GCC)^36^. The same study also demonstrated a strong positive correlation between myelin water fraction (MWF) and LFB staining. Complementary MRI studies of MWF further support our findings, converging on the consensus that the SCC, GCC, and IC are among the most myelin-rich regions of the brain^37–39^.

Our findings in human participants are further supported by recent preclinical validation from our group, in which tNOE demonstrated the highest sensitivity to myelin among several candidate CEST contrasts in a rodent model of demyelination^40^. In that study, tNOE contrast strongly correlated with histological markers of myelin (Luxol Fast Blue, MBP immunostaining), confirming that the observed tNOE reductions are mechanistically linked to myelin loss rather than nonspecific tissue alterations. This cross-validation between preclinical histology and in vivo human imaging strengthens the biological specificity of tNOE as a myelin-sensitive biomarker and underscores its translational potential for monitoring demyelinating diseases.

A central finding is the sensitivity of tNOE contrast to regional lipid alterations in MS. Compared with healthy controls, MS subjects exhibited markedly reduced tNOE signal in lesions, consistent with the well-established loss of lipid-rich myelin. Importantly, reductions were also observed in non-lesional regions, such as the splenium, suggesting that tNOE may capture diffuse demyelination or microstructural abnormalities that are not apparent on conventional MRI. This aligns with histopathological evidence that MS pathology extends beyond focal lesions, affecting normal-appearing white matter through mechanisms such as axonal transection, gliosis, and chronic microglial activation^41^ By detecting these subtler lipid changes, tNOE could provide an earlier and more sensitive marker of disease burden than current clinical imaging methods. In addition, tNOE shows lower overall GM contrast in MS subjects than in healthy controls.

The tNOE_MTR_ metric in the normal-appearing white matter (NAWM) of MS patients was significantly altered across all major white matter regions compared to healthy participants, with the splenium of the corpus callosum showing the largest differences of 36%. This may suggest a loss of myelin-associated lipids and other lipids in that region and aligns with the findings of Laule et al.,^42^ who reported a 13% lower myelin water fraction (MWF) in the splenium of the corpus callosum of MS subjects relative to healthy controls. Using ssNOE-related imaging, Jacobs et al.^43^ reported myelin changes in normal-appearing WM and GM of MS patients, though individual regional variation did not reach statistical significance. Further, we demonstrated excellent reproducibility on both inter- and intra-day basis. These findings underscore the potential of tNOE mapping to probe regional variability in lipid composition and white matter integrity during normal aging and in neurological disorders.

Catalytic autoantibodies with proteinase activity against myelin basic protein (MBP) are a distinct feature of several autoimmune disorders ^44^. These autoantibodies, known as abzymes (Abz), combine both antibody and proteinase activity within a single molecule. Abzymes targeting MBP, referred to as MBP Abz, are commonly detected in the serum of individuals with MS^45–49^. MBP Abz are produced due to myelin compaction loss, which occurs when the enzyme peptidylarginine deiminase deaminates arginine residues in MBP, altering its charge. This process exposes a normally hidden surface of the MBP to T cells, thereby triggering an autoimmune response. A substantial body of evidence indicates that MBP Abz plays a significant role in the pathogenesis of MS^50,51^. Another study has shown that anti-MBP antibody-mediated proteolytic activity significantly correlated with scores on the MS Expanded Disability Status Scale (EDSS)^50^. The significant changes in tNOE contrast observed in the brains of MS patients may be linked to the presence of MBP Abz. Further research is needed to establish correlations among antibody concentration, alterations in tNOE contrast, and EDSS.

Beyond MS, the implications of this work extend to other neurodegenerative conditions in which lipid metabolism plays a central role. In Alzheimer’s disease, altered phospholipid homeostasis and myelin breakdown have been linked to cognitive decline^52^. Similarly, in leukodystrophies and traumatic brain injury, disrupted lipid integrity contributes to progressive neurodegeneration^53^. The high reproducibility of our protocol suggests it could serve as a platform technology for tracking lipid-related changes across a spectrum of diseases.

Compared to ssNOE-derived rNOE imaging, the tNOE approach offers significant advantages for clinical translation. In contrast to the long 1-3s duration of saturation pulses used in ssNOE, the shorter duration of adiabatic inversion pulses as used in tNOE imaging reduces contributions from bound and bulk water to the tNOE signal vs. the ssNOE signal and thereby further enhances the specificity for aliphatic proton signals from lipids and proteins. The use of adiabatic inversion pulses ensures B1 robustness and eliminates the need for B_1_ calibration, which may be required for ssNOE-based mapping to handle B_1_ inhomogeneities at a higher-field (7T) human MRI scanner. This enables faster scan times (~ 3 minutes) without sacrificing this specificity. Importantly, while ssNOE-derived MTR maps sometimes may not be able to capture regional variability within WM tracts faithfully and may even show higher signals in some peripheral regions, tNOE mapping revealed stronger contrast in densely myelinated areas such as the splenium, corpus callosum, internal capsule, and posterior thalamic radiation, with lower signals near the cortex (Fig. 5 of Kumar et al.^25^).

Several limitations warrant consideration. This proof-of-concept study was limited to a small cohort of MS patients. Larger studies are needed to confirm sensitivity and to explore correlations with clinical disability and cognitive outcomes. Future work should also compare tNOE with other established myelin markers, such as myelin water imaging, magnetization transfer ratio, and quantitative susceptibility mapping, to further validate its pathological specificity. Nonetheless, we have demonstrated that tNOE MRI has the potential to become a transformative marker for diagnosing and monitoring lipid-related changes in healthy aging and neurodegenerative diseases such as MS.

In summary, 3D tNOE-MRI with adiabatic pulses provides a lipid-sensitive, reproducible contrast mechanism capable of detecting both focal and diffuse myelin pathology in MS. By bridging biochemical specificity with clinical feasibility, tNOE-MRI has the potential to advance our understanding of neurodegenerative disease mechanisms and to improve early detection and therapeutic monitoring. Future work will focus on expanding its application to larger cohorts, exploring its utility in disease models, and further refining the protocol for both 3T and 7T MRI to enhance its clinical translation.

## Figures and Tables

**Table 1:** Summary of inter- and intra-day repeatability analysis for tNOE measurements across five subjects in the WM and GM. In most subjects, the coefficients of variation (COVs) for both inter-day and intra-day scans are below 5%, demonstrating the reliability of the optimized protocol for clinical and research applications.

	Inter-day scans				Intra-day scans					
	*Subject 1*		*Subject 2*		*Subject 3*		*Subject 4*		*Subject 5*	
	WM	GM	WM	GM	WM	GM	WM	GM	WM	GM
**Scan 1 (%)**	14.96	9.19	14.19	9.65	17.37	9.11	14.97	9.72	17.15	9.45
**Scan 2 (%)**	14.30	8.79	15.25	9.98	17.60	9.01	14.42	9.45	17.43	9.69
**COV (%)**	2.48	3.21	5.06	2.34	0.91	0.77	1.82	1.91	1.11	1.81

## Data Availability

The datasets generated and/or analyzed during the current study are available from the corresponding author on reasonable request.
